# Potassium deficiency induces the biosynthesis of oxylipins and glucosinolates in *Arabidopsis thaliana*

**DOI:** 10.1186/1471-2229-10-172

**Published:** 2010-08-11

**Authors:** Stephanie Troufflard, William Mullen, Tony R Larson, Ian A Graham, Alan Crozier, Anna Amtmann, Patrick Armengaud

**Affiliations:** 1 Faculty of Biomedical and Life Sciences, University of Glasgow, Glasgow G12 8QQ, UK; 2 Centre for Novel Agricultural Products, Department of Biology, University of York, PO BOX 373, York YO10 5YW, UK; 3 Current Address: Institut Jean-Pierre Bourgin, UMR1318 INRA AgroParisTech, Centre de Versailles, RD10, 78026 Versailles Cedex, France

## Abstract

**Background:**

Mineral fertilization and pest control are essential and costly requirements for modern crop production. The two measures go hand in hand because plant mineral status affects plant susceptibility to pests and *vice versa*. Nutrient deficiency triggers specific responses in plants that optimize nutrient acquisition and reprogram metabolism. K-deficient plants illustrate these strategies by inducing high-affinity K-uptake and adjusting primary metabolism. Whether and how K deficient plants also alter their secondary metabolism for nutrient management and defense is not known.

**Results:**

Here we show that K-deficient plants contain higher levels of the phytohormone jasmonic acid (JA), hydroxy-12-oxo-octadecadienoic acids (HODs) and 12-oxo-phytodienoic acid (OPDA) than K-sufficient plants. Up-regulation of the 13-LOX pathway in response to low K was evident in increased transcript levels of several biosynthetic enzymes. Indole and aliphatic glucosinolates accumulated in response to K-deficiency in a manner that was respectively dependent or independent on signaling through Coronatine-Insensitive 1 (COI1). Transcript and glucosinolate profiles of K-deficient plants resembled those of herbivore attacked plants.

**Conclusions:**

Based on our results we propose that under K-deficiency plants produce oxylipins and glucosinolates to enhance their defense potential against herbivorous insects and create reversible storage for excess S and N.

## Background

Application of fertilizers and pesticides is common practice in modern agriculture to ensure the growth and health of crops. Both measures present substantial costs not only to food production but also to the environment. The need to cater for a rapidly growing world population means that over the next decades food production will have to become more cost effective and expand into areas that are naturally poor in mineral nutrients. It is therefore essential to obtain now a better understanding of how plants adapt to mineral deficiencies and how mineral deficiency impacts on crop susceptibility to pests.

Metabolomic approaches have enabled the determination of metabolite profiles of plants grown in specific conditions of nitrogen (N), phosphorus (P), sulfur (S) or potassium (K), and these have been linked to transcript profiles in an effort to unravel the regulatory network adjusting individual metabolic pathways to mineral nutrient supply [1]. Clearly, the observed changes in metabolite contents affect the nutritional quality of the plant for any animal or microorganism feeding on it. For example, sub-optimal supply of K to plants leads to a characteristic increase in the concentrations of sugars and amino acid that could increase the attractiveness of K-deficient plants for herbivorous insects [2,3]. By correlating metabolite profiles of *Arabidopsis thaliana *plants grown in varying K conditions with transcript profiles and post-translational enzyme activities we identified specific changes in primary metabolic and regulatory pathways that occur upstream and downstream of the observed changes in sugar levels [2].

Many secondary metabolites produced by plants act as toxins and deterrents for pests and pathogens. Since they are often rich in N and/or S deficiency in N or S impacts on the biosynthesis of these compounds and hence on the defense potential of plants [4,5]. A link between K supply, secondary metabolism and defense is less obvious. Nevertheless, a microarray analysis of *A. thaliana *plants carried out in our laboratory revealed that many of the transcripts that were reversibly changed by K-deficiency were linked to the plant hormone jasmonic acid (JA) [6]. JA and its derivates play an important role in plant responses to wounding, herbivores and pathogens [7,8]. JA biosynthesis occurs through the octadecanoid acid pathway, which starts from the oxidation of polyunsaturated fatty acids by lipoxygenase and produces a number of intermediate oxylipins [9,10]. Several of these have signal function and antimicrobial properties [11]. JA signaling employs the E3 ubiquitin ligase SCF^COI1 ^complex that targets transcriptional repressors of JA target genes for degradation through the 26 S proteasome [12-17]. A central component of this complex is the F-box protein COI1 (Coronatine-Insensitive 1), which binds directly to JA-Ile thereby acting as a JA receptor [15]. Our previous finding that a large part of the K-dependent transcriptome is absent or replaced in *A. thaliana coi1-*16 mutants is therefore further support for a role of JA in plant responses to K-supply [18].

Transcript profiles of K-deficient plants also showed considerable overlap with transcript profiles of plants exposed to herbivorous insects [18,19]. One well known JA-induced defense mechanism against herbivorous insects involves the myrosinase-glucosinolate system [20]. After tissue damage myrosinases are released from cell compartments and hydrolyze glucosinolates (GLS) to generate bioactive substances such as isothiocyanate, thiocyanate and nitrile compounds that are toxic for insects and microbial pathogens [21-24]. Increasing interest in GLS and their degradation products is due to their potential as human cancer-prevention agents, crop-protection compounds and biofumigants in agriculture [25,26]. GLS are sulfur rich compounds derived from amino acids and - apart from their function in defense - they also act as a reversible S-store [5]. Similarly, vegetative storage protein (VSP), which is also up-regulated in a COI1-dependent manner under K-deficiency, is an important N store and plays a role in plant defense against pests [18,27].

In this study we have further investigated the effects of K-starvation on the biosynthesis of oxylipins and GLS in *A. thaliana *plants. GLS levels were measured both in wildtype and in *coi1*-16 mutants to establish their dependence on COI1-signaling. We found that besides JA other oxylipins with potential signal function accumulate in K-deficient plants, and that K-deficiency induces the production of indole and aliphatic GLS through COI1-dependent and COI1-independent pathways respectively. The results are discussed in the context of previous results from our group concerning changes of primary metabolism and herbivore susceptibility in K-deficient plants [2,18].

## Results

### Oxylipin concentrations rise in response to low K

**Figure 1:**
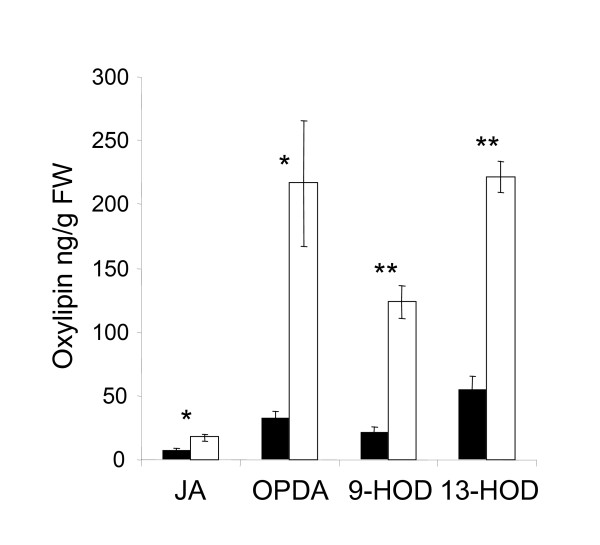
**K deficiency increases the net production of several oxylipins**. Oxylipin concentrations in shoots of control and K-deficient plants. Plants were grown from germination for 2 weeks on vertical Petri dishes in control (black) or K-free medium (white). Averages (± SE) of oxylipin concentrations from three independent experiments are shown. In each experiment oxylipins were extracted from shoot tissue pooled from approx. 75 plants, and analysed by LC-MS. Stars indicate significant differences between control and K-starved plants at p < 0.05 (*) or p < 0.01 (**) as determined by t-test. JA: Jasmonic acid; OPDA: 12-oxo-phytodienoic acid; 9-HOD: 9-hydroxy-12-oxo-octadecadienoic acid; 13-HOD: 13-hydroxy-12-oxo-octadecadienoic acid.

Using liquid chromatography-mass spectrometry (LC-MS) [28] we identified JA and several other oxylipins in leaves of *A. thaliana*. Levels of 9-hydroxy-12-oxo-octadecadienoic acid (9-HOD; γ-ketol), 13-HOD (α-ketol), 12-oxo-phytodienoic acid (OPDA) and jasmonic acid (JA) were significantly higher in K-starved than in K-sufficient plants (Fig. 1). The largest increase was found for OPDA (> 5-fold), the smallest for JA (1.8-fold). K deficiency also affected the ratio of 13-/9-HOD (1.8 in K-deficient plants *vs*. 2.7 in control plants). Within 24 h of K re-supply oxylipin levels decreased again and the 13-/9-HOD ratio returned towards the control value (Table 1).

**Table 1:** 13/9-HOD ratios and oxylipin concentrations after 24 h K re-supply

	Control	K-deficient	24 h K re-supply	
13-HOD/9-HOD ratio	2.7 ± 0.15	1.8 ± 0.08	2.2 ± 0.09	

	**JA**	**OPDA**	**9-HOD**	**13-HOD**
Concentration (%)^1 ^after 24 h K re-supply	53.0 ± 19.9	48.2 ± 23.6	58.9 ± 20.9	69.0 ± 22.6

^1^In % of concentration in plants re-supplied with K-free medium.

### K-deficiency induces *VSP*, *LOX2 *and other enzymes in the 13-LOX pathway

**Figure 2:**
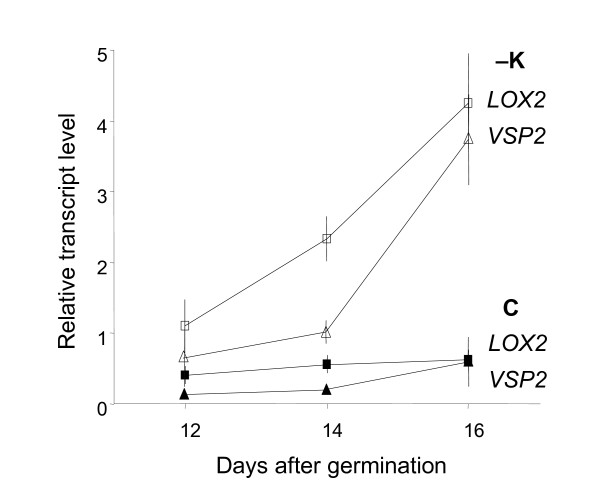
**Induction of *LOX2 *and *VSP2 *in response to K deficiency**. Quantitative PCR analysis of *LOX2 *(squares) and *VSP2 *(triangles) in shoots *A. thaliana *plants grown from germination for 12 to 16 days on vertical Petri dishes containing control medium (C, black symbols) or K-free medium (-K, open symbols). Approximately 50 plants were pooled for RNA isolation. Bars show averages ± standard error of technical replicates, normalised to the expression level of the constitutive gene *YLS8 *(see Material and Methods for qPCR details and primers). Invisible error bars are smaller than symbols.

**Figure 3:**
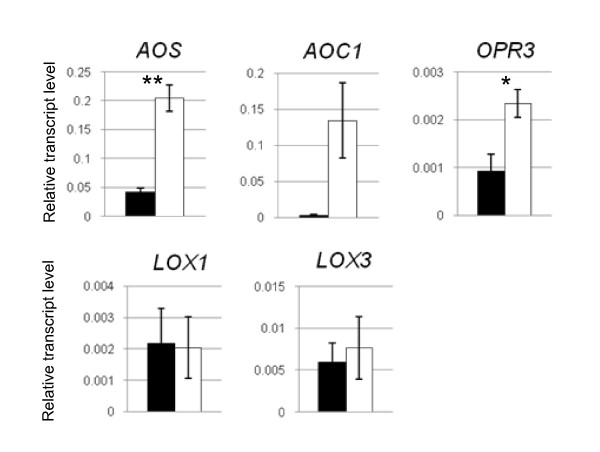
**Response of oxylipin biosynthetic genes to K deficiency**. Quantitative PCR analysis of selected oxylipin biosynthesis genes in shoots of *A. thaliana *plants grown for 14 days on vertical Petri dishes containing a control medium (black) or a K-free medium (white). Approximately 50 plants were pooled for RNA isolation. Bars show averages ± standard errors of technical replicates, normalised to the expression level of the constitutive gene *EF1α *(see Material and Methods for qPCR details and primers). Stars indicate significant differences between control and K-starved plants at p < 0.05 (*) or p < 0.01 (**) as determined by t-test.

Previous microarray experiments indicated reversible induction by low K of genes closely related to JA [6], particularly *VSP2 *(At5g24770, [29]), a well known target of JA-signaling, and *LOX2 *(At3g45140) encoding a 13(S)-lipoxygenase (LOX), which catalyses the initial step of JA production [30]. We monitored the time course of this induction over progressing K-deficiency and found that both genes are already induced at day 12 (before visible symptoms appear) and experience a further sharp rise in transcription over the following days (Fig. 2). Thus induction of JA biosynthesis and signaling mirrors (or slightly precedes) changes of primary metabolite contents in the shoots (compare with Fig. 1 in [2]). Further qPCR experiments showed that transcripts for biosynthetic enzymes downstream of LOX2, allene-oxide synthase (*AOS*, At5g42650), allene-oxide cyclase (*AOC1*, At3g25760) and 12-oxophytodienoate reductase (*OPR3*, At2g06050), are also up-regulated in K-deficient plants (Fig. 3). By contrast, *LOX1 *(At1g55020) encoding a 9-LOX and *LOX3 *(At1g17420) encoding another isoform of 13-LOX did not show significant changes. Additional file 1 shows microarray results for other genes with putative function in oxylipin biosynthesis, none of which showed significant changes of transcript levels in response to K-deficiency or re-supply.

**Figure 4:**
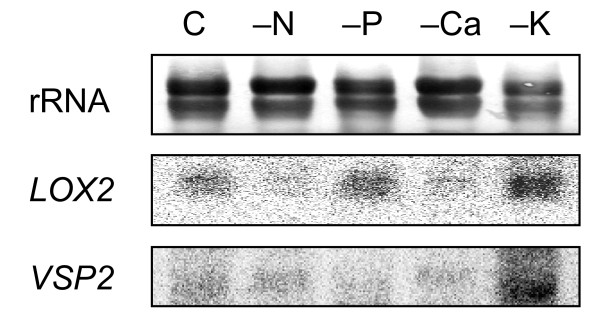
**Transcript levels of JA marker genes in N, P and Ca deficient plants**. Northern blot analysis of *LOX2 *and *VSP2 *transcript level in response to different nutrient deficiencies. Plants were grown for 14 days on vertical Petri dishes containing a control medium (control, C) or media with low levels of nitrogen (-N), phosphorus (-P), calcium (-Ca) or potassium (-K) (see Additional files 2 and 3 for media composition and plant appearance). *LOX2 *and *VSP2 *signals were obtained after hybridization with the corresponding ^32^P-labelled probes and autoradiograms are presented. rRNA levels after a methylene blue staining show the total amount of RNA in each sample blotted onto the membrane. Note that due to the strong accumulation of *LOX2 *and *VSP2 *transcripts in low-K plants less RNA was loaded onto the gel.

To investigate whether increased oxylipin biosynthesis is a general feature of nutrient deficient plants we measured transcript levels of *LOX2 *and *VSP2 *in shoots of 2-weeks old plants grown on media that lacked nitrogen (N), phosphorus (P) or calcium (Ca) (for growth media see Additional file 2). As previously done for K, the specific nutrient concentration in the growth medium was adjusted so that it caused after a growth period of 2 weeks clear but non-lethal deficiency symptoms (e.g. smaller and chlorotic shoots; Additional file 3). As shown in Fig. 4 we detected no increase in *LOX2 *or *VSP2 *transcripts in plants grown in low N, P or Ca for 14 days compared to control plants while a strong increase was again observed in K-deficient plants (Fig. 4).

### K deficiency increases total glucosinolate levels and alters GLS profiles

**Figure 5:**
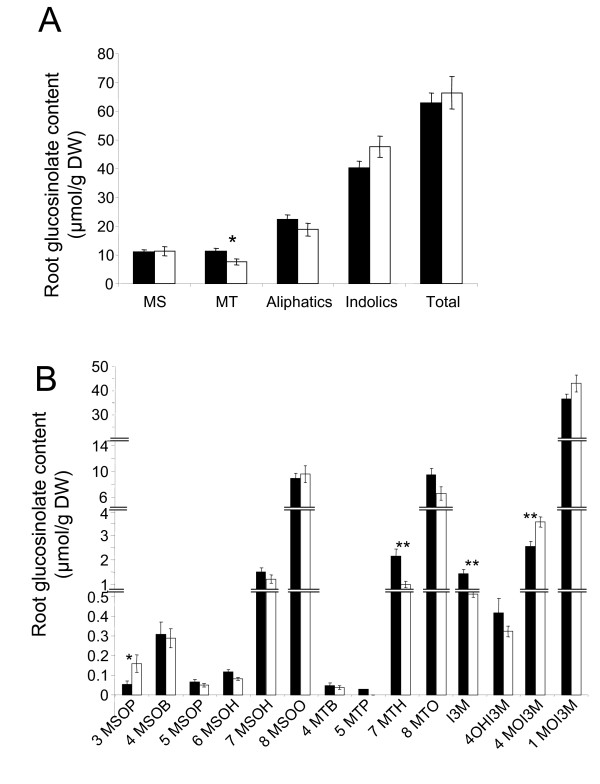
**Root glucosinolate profiles in control and K-deficient wildtype plants**. Glucosinolate profiles in roots of wildtype (Col0) *A. thaliana *plants grown in vertical Petri dishes in control (black) and K-deficient conditions (white). A. Glucosinolates grouped into methionine-derived aliphatic GLS (sub-divided into methylsulfynylalkyl (MS) and methylthiolalkyl (MT) compounds), tryptophan-derived indole GLS and total GLS (sum of aliphatic and indole GLS). B. Individual GLS. Note that a scale shift was introduced on the y-axis to visualize differences between control and low-K conditions for all compounds. Values are averages of three biological replicates measured in three technical replicates. Bars show standard errors, asterisks indicate the significance of a difference between control and K-starved plants (* for p < 0.05, ** for p < 0.01). Full names of all glucosinolates are given in the Additional file 4.

**Figure 6:**
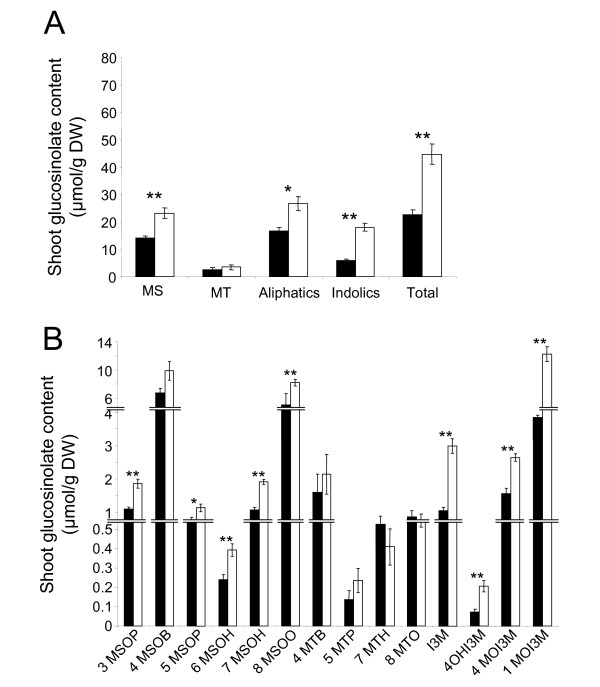
**Shoot glucosinolate profiles in control and K-deficient wildtype plants**. Glucosinolate profiles in shoots of wildtype (Col0) *A. thaliana *plants grown in vertical Petri dishes in control (black bars) and K-deficient conditions (white bars). For details see Fig. 5.

**Figure 7:**
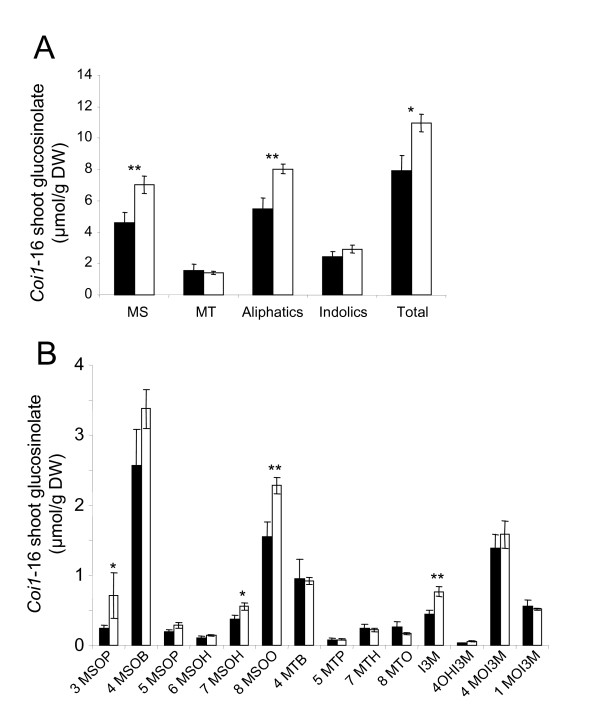
**Shoot glucosinolate profiles in control and K-deficient *coi1*-16 plants**. Glucosinolate profiles in shoots of *A. thaliana coi1*-16 plants grown in vertical Petri dishes in control (black bars) and K-deficient conditions (white bars). For details see Fig. 5.

Considering the importance of glucosinolates (GLS) in plant-herbivore interactions and nutrient management we carried out an LC/MS^2 ^analysis of leaf tissue from K-sufficient and K-deficient *A. thaliana *Col0 and *coi1-16 *plants and identified 14 different GLS species (Figs. 5, 6 and 7; for names and abbreviations see Additional file 4). Ten of these belong to the class of aliphatic GLS comprising methylsulfinylalkyl compounds (MS-GLS) and their methylthiolalkyl precursors (MT-GLS). The remaining four were indole (tryptophan-derived) GLS. Total GLS concentrations in roots and shoots of control plants were 62.8 ± 3.54 and 22.5 ± 1.83 μmoles (g DW)^-1 ^respectively (black bars in Figs. 5A and 6A). In the roots 1MOI3 M comprised 57% of total root GLS (Fig. 5B) thereby making indole GLS the larger class in this organ (64% of total root GLS). In the shoot aliphatic GLS represented 75% of the total GLS with 4MSOB being the predominant compound (Fig. 6B).

Plants subjected to K deficiency displayed significantly altered GLS profiles. In the roots, the total concentrations of the two main GLS classes were little affected by K deficiency although MT-GLS pools showed some depletion (Fig. 5A). However, root concentrations of individual GLS were significantly higher (3MSOP and 4MOI3M) or lower (7MTH and I3M) in K-deficient plants than in control plants (Fig. 5). In the shoots, total GLS levels were approximately two times higher in K-deficient plants than in control plants with indole GLS increasing more strongly than aliphatic GLS (Fig. 6A). Among the aliphatic GLS, K-deficiency induced accumulation was apparent for MS-GLS but not for MT-GLS indicating rapid oxidation of the precursors. The increase of total shoot GLS content was based on significant increases in several individual compounds, particularly 1MOI3 M (Fig. 6B, note different scales of the y-axes).

### K-dependent changes in indole GLS are abolished in *coi1*-16 mutants

Analysis of shoot samples from plants grown in control conditions revealed considerably lower GLS levels in *coi1*-16 than in wild type plants for all major GLS classes (Fig. 7A, cf. Fig. 6A). *coi1*-16 plants grown in low K still experienced a significant increase in shoot GLS content but to a lesser extent than wildtype plants (1.3-times instead of 2-times). Most notably, the strong accumulation of indole GLS observed in K-deficient wildtype plants did not occur in K-deficient *coi1*-16 plants. K deficiency-induced increases in individual indole GLS were either reduced (e.g. I3M) or completely abolished (e.g. 1MOI3M) in the mutant (Fig. 7B, cf. Fig. 6B). We conclude that both the basal net production of indole GLS and its up-regulation in response to low K require an intact JA-COI1 signaling pathway. The remaining increase of total GLS level in K-deficient *coi1*-16 shoots originated from an increase of MS-GLS compounds, which displayed similar relative changes as in wildtype although absolute levels were much lower. This indicates that the basal net production of aliphatic GLS production is dependent on JA-COI1-signaling but its up-regulation during K deficiency is not.

### K deficiency induces genes with function in glucosinolate metabolism

**Figure 8:**
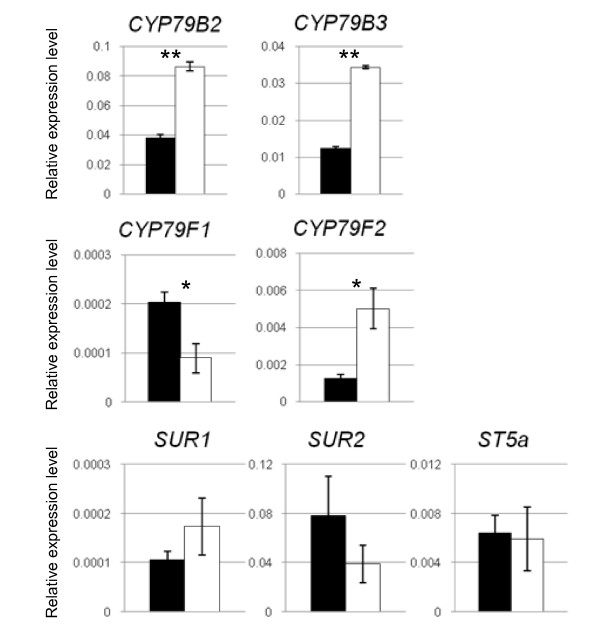
**Response of glucosinolate biosynthetic genes to K deficiency**. Quantitative PCR analysis of transcript levels of glucosinolate biosynthesis genes in *A. thaliana *plants grown for 14 days on vertical Petri dishes containing control medium (black) or -K medium (white). Approximately 50 plants were pooled. Averages and standard error of technical replicates are shown, normalised to the expression level of the constitutive gene *EF1alpha *or *YLS8 *(see Material and Methods for qPCR details and Additional file 5 for primers). Stars indicate significant differences between control and K-starved plants at p < 0.05 (*) or p < 0.01 (**) as determined by t-test.

The list of K-dependent transcripts identified in previous microarray experiments contained a number of genes with putative function in the biosynthesis (CYP and MAM families) or breakdown (myrosinase family) of GLS [6]. Cytochromes P450 encoded by *CYP79B2 *(At4g39950) and *CYP79B3 *(At2g22330) catalyse the first step in the biosynthesis of tryptophan-derived indole GLS [31,32]. qPCR confirmed that transcript levels of both enzymes were increased in K-starved plants (2- and 3-fold compared to control plants, Fig. 8). *CYP79F1 *(At1g16410) and *CYP79F2 *(At1g16400), encode enzymes that catalyse the synthesis of short-chain and long-chain elongated methionine-derived aliphatic GLS [33]. For these two genes we found contrasting transcript responses to K-deficiency; CYP79F1 is down-regulated while CYP79F2 is up-regulated (Fig. 8). Transcripts for enzymes catalyzing GLS biosynthesis steps downstream of CYP79 s such as SUR2 (CYP83B1; At4g31500, [34]), SUR1 (C-S lyase, At2g20610, [35]) and the sulfotransferase ST5a (At1g74100, [36]) did not show significant changes (Fig. 8).

## Discussion and Conclusions

Dissecting pathways at the crossroads of biotic and abiotic stress responses of plants is a new and exciting research area that should lead to integrated strategies for fertilizer and pesticide usage in the context of sustainable agriculture. Based on previous analyses of transcriptional responses to K-deficiency and re-supply, the objective of this study was to investigate whether and how plant K status affects the production of signal compounds and secondary metabolites that are important for nutrient management and plant defense.

### Oxylipin production and signaling in K-deficient plants

We have shown here that the levels of several oxylipins increase in K-starved plants (Fig. 1). The measured 1.8-fold increase of shoot JA in K-deficient plants is somewhat lower than the 3-fold increase determined by Cao et al. [37] using identical growth conditions, which is likely to be due to the difference in the sampled tissues (entire shoot vs. fourth leaf respectively). While JA (in its biologically active form JA-Ile [38]) is considered the most important signal compound deriving from 13-LOX pathway there is increasing evidence that other oxylipins act as signaling compounds in their own right. The observed (> 5 times) increase in OPDA level during K-deficiency is particularly interesting because the transcript profile of K-deficient plants overlaps strongly with that of OPDA-treated plants ([18], Supplemental material SI4A therein). OPDA has been reported to function as a signal in its own right during plant defense and wounding responses, acting through both COI1-dependent and COI1-independent pathways [10,39,40]. Our recent analysis of COI1-dependence of K-responsive genes indicates that the same is true here, but we identified considerably more OPDA-responsive genes among COI1-dependent than among COI-independent K-responsive genes ([18], Supplemental material SI4B-E therein). COI1-defective mutants will therefore not readily distinguish between JA and OPDA.

We also found increased levels of HOD in K-deficient plants (Fig. 1). Experiments with pure oxylipins [41] showed that treatments with 9- and 13-HOD cause root phenotypes (waving and arrest of lateral root growth) that differ from those caused by OPDA and JA treatments (loss of apical dominance and increased numbers of lateral roots), indicating a specific function of these oxylipins in plant development. Interestingly, the root phenotype of K-deficient plants (characterized by lateral root growth arrest, [6]) resembles the HOD-induced phenotype suggesting that the observed rise in HOD-levels could be responsible for some of the K-deficiency symptoms. Analysis of a number of mutations interrupting the biosynthetic pathway at different positions is now required to elucidate specific roles of individual oxylipins in plant responses to K stress.

The measured increase of transcript levels of the biosynthetic enzymes LOX2, AOS, AOC and OPR3 under K-deficiency (Fig. 2, 3) indicates that the K-dependent production of oxylipins in the 13-LOX pathway is under transcriptional control. The measured relative changes of the individual compounds (e.g. OPDA/JA) are also partly matched by the transcript levels of the respective biosynthetic enzymes (e.g. higher levels and increases of AOS and AOC than OPR3) but will ultimately depend on the relative pool sizes of the individual oxylipins and the rates of all enzymatic and non-enzymatic reactions that contribute to their production, modification and decay [42]. The observed increase in 9-HOD is surprising because transcripts of enzymes in the 9-LOX pathway are unchanged in K-deficient plants (e.g. *LOX1*, Fig. 3 and Additional file 1, [43]). It is therefore likely that this increase is due to direct (probably non-enzymatic) conversion from other oxylipins rather than transcriptional up-regulation of the 9-LOX pathway.

Importantly, the changes of oxylipin levels are reversed by short-term K re-supply (as previously also shown for changes in transcripts, tissue K contents and most primary metabolites [2,6], Table 1) proving that they are not secondary effects of leaf senescence or other irreversible symptoms of K-deficiency. Previous studies by other groups indicated that in some cases S-deficiency also induces the 13-LOX pathway (e.g. up-regulation of *LOX2*, *OPR1 *(At1g76680) and/or *AOS *[44-46]), and we therefore tested the response of *LOX2 *and *VSP2 *to a number of other nutrients. After 2 weeks growth in nutrient limiting conditions (see Additional file 2 for growth media) the plants did not show any indication for transcriptional responses of *LOX2 *or *VSP2 *to N, P or Ca deficiency (Fig. 4), despite the fact that they exhibited clear deficiency symptoms (see Additional file 3). In this context it is worth noting that, unlike other nutrients, external K directly modulates factors that are important for early responses during wounding and pathogen responses such as membrane potential, transmembrane K/Ca fluxes and cytoplasmic Ca [47,48]. The observed link between K-supply and *LOX2 *therefore provides a new opportunity to identify upstream components of oxylipin signaling.

### Glucosinolate contents and profiles in K-deficient plants

Oxylipin signaling plays an important role in plant defense against a wide range of specialist and non-specialist herbivorous insects [48]. (Me-)JA application enhances plant immunity and this effect is at least in part due to induced production of secondary metabolites such as glucosinolates [49]. For example, pre-exposure to JA reduces numbers and development of thrips (*Frankliniella occidentalis*) on *A. thaliana *and *Brassica rapa *plants [50]. We have recently reported that pre-exposure to low-K conditions also decreases thrips damage of *A. thaliana *plants [18]. It is tempting to hypothesize that this effect is based on oxylipin-induced production of glucosinolates. However, protection against thrips by low K status was still evident in *cyp79b2/b3 *mutants, which are defective in the production of indolic glucosinolates [51]. To further investigate this issue we measured here 14 different GLS species in control and K-starved plants (wildtype and *coi1-16*) including short-chain and long-chain aliphatic GLS and their precursors.

The root and shoot GLS profiles that we determined for control plants are in good agreement with previous reports [51-53]. We found that root GLS levels are generally higher than shoot levels, dominated by the indole 1MOI3 M and little affected by K deficiency. It is noteworthy that the lack of responsiveness in root GLS, previously observed for other stresses [53], is also apparent for a stress that originates in the roots [2]. This suggests not only different physiological functions of GLS in roots and shoots but also that a role of JA-signaling during K-deficiency is restricted to the shoot (where 1MOI3 M is sensitive to K and COI1, see Figs. 5 and 6). Shoot GLS profiles of K-deficient plants resembled those of plants subjected to MeJA treatment or herbivory [21,54,55]. For example, indole GLS are the most responsive class of GSLs with approx. 3-times increases of I3 M, 4OHI3 M and 1MOI3 M contents. At least for 4OHI3 M and 1MOI3 M, this accumulation is clearly a consequence of the K-induced increase in JA as it no longer occurs in *coi1*-16 plants. Methylsulfynylalkyl compounds were also higher in K-starved than in control plants but this increase also occurs in *coi1-*16 mutants. A similar COI1-independent increase in aliphatic GLS was previously reported for plants exposed to herbivorous lepidopterae [55].

The observed increase of indole GLS in K-deficient plants corresponds to transcriptional up-regulation of CYP79B2 and CYP79B3 (Fig. 7), the enzymes that catalyze the first step of GLS biosynthesis from tryptophan [31,32]. As in the case of MeJA treatment [51], application of bacterial elicitors and herbivory [21,55] this response requires an intact COI1 (see Supplemental Material SI5 in [18]). Synthesis of aliphatic GLS from chain-elongated methionine is catalyzed by enzymes encoded by *CYP79F1 *and *CYP792*, which have different but overlapping specificity and expression patterns within the plant. The observed opposite change of the transcript levels of these two enzymes (Fig. 7) is interesting but the overall and specific effects on aliphatic GLS are difficult to predict. The signal (or substrate) linking K-deficiency to the biosynthesis of aliphatic GLS and its enzymatic targets remain to be identified.

### Putative benefits of oxylipin and GLS accumulation under K-deficiency

The similarity of the changes of transcripts, oxylipins and glucosinolates observed in K-deficient plants and in plants subjected to herbivorous insects suggests that induction of the JA-pathway under K-deficiency has evolved as a means to increase the plant's defense potential. Both VSP and GLS act as deterrents for non-specialist herbivorous insects [26,56] and K-deficient plants are indeed more resistant to thrips [18]. A 'prophylactic' defense response makes sense considering that K-deficient plants display a number of features that makes them more attractive for herbivorous insects (e.g. increased levels of sugars and amino acids, less rigid cell walls etc.; [3]), and more sensitive to leaf damage (smaller leaf surface). However, our previous experiments with *cyp79b2/b3 *mutants showed that increased thrips resistance of K-deficient plants does not require indole GLS [18]. Whether the observed increase in aliphatic GLS is critical for thrips resistance should now be investigated using *cyp79f1/f2 *mutants.

Susceptibility of plants to herbivorous insects is closely related to nutrient management (chemical and physical allocation of C, N and S). For example it has been shown that JA-dependent re-allocation of VSP from shoots to roots is part of the plant defense against herbivorous pests [27,57,58]. Thus an alternative benefit of the observed changes could be that induction of VSP and GLS production assists the plant in managing a nutrient imbalance that occurs as a result of K-deficiency. Our previous detailed analysis of primary metabolism in *A. thaliana *plants indicated direct inhibition of root glycolysis by low K resulting in a situation of N and S surplus, which is apparent for example in an accumulation of glutamine and tryptophan in the shoots [2]. The measured time course of *LOX2 *and *VSP2 *transcription (Fig. 2) is in good agreement with the time course of metabolite changes in the shoots [28]. Most notably glutamine and tryptophan start to accumulate from day 12 or 14 onwards in the shoots of K-deficient plants (cf. Fig. 1 in [2]). Thus, JA-induced production of VSP could provide a means to accommodate excess N, and increased production of GLS could create a sink for surplus S under K-limiting conditions [25-27]. This hypothesis is supported by the finding that low S-supply causes opposite changes; depletion of tryptophan and decrease of GLS [5].

Because production of indole GLS shares the same pathway as auxin production GLS metabolism also impacts on auxin levels [59,60]. In plants grown in low-S conditions, a decrease in GLS levels decrease is accompanied by an increase in lateral root formation/elongation [61]. This phenotype has been suggested to be due to high auxin levels originating from an increase of indole acetonitrile (a GLS breakdown product and auxin precursor) together with increased nitrilase activity [62]. Again, K-deficient plants display the opposite features; lateral root growth is arrested [6] and auxin levels decrease [63]. However, a direct link between GLS, auxin and root development it is difficult to reconcile with our finding that levels of indole GLS in the roots are not changed by K-deficiency. A GLS-linked auxin signal in K-deficient plants is therefore likely to be shoot-derived.

In summary, based on the results presented here and in previous papers we propose that induction of JA-biosynthesis in K-starved plants triggers the production of compounds that accommodate surplus N and S, with the additional benefits of limiting food supply for herbivorous insects and presenting deterrents and toxins. Our findings call for a detailed investigation into the effects of varying nutrient ratios (K/N/S) on plant secondary metabolism, root development and defense.

The established link between plant K status and GLS biosynthesis has also important implications for biotechnological efforts to manipulate GLS production for dietary and medical purposes [25,64]. Our study suggests that fine-tuning of K/S/N ratios in the fertilizer will be critical for maximizing total GLS production and manipulating GLS profiles.

## Methods

### Plant material and growth conditions

*Arabidopsis thaliana *(Col0 wildtype or *coi1*-16) plants were grown on sterile vertical agar plates or hydroponically as described previously [6,65]. The composition of the nutrient media is given in the Additional file 2. For long-term starvation, plants were grown on agar plates for 2 weeks. K-starved plants were subjected to short-term (6-24 h) K re-supply by replacing the condensed solution at the bottom of the plate with 5 ml of liquid 'K-free' medium supplemented with 10 mM KCl (K re-supply) or not (re-supply control).

### Extraction and LC/MS analysis of oxylipins

Shoots of plants grown in control and K-deficient conditions were pooled from at least five plates (> 75 individual plants), frozen and ground in liquid nitrogen. 200 mg ground tissue from each sample were used for extraction and quantification of oxylipins with HPLC tandem mass spectrometry according to a previously published protocol [28].

### Northern blot analysis

Total RNA was extracted using Trizol (Invitrogen). Northern blotting was performed following standard protocols [66]. Probes for *LOX2 *and *VSP2 *were amplified by PCR (for primers see Additional file 5), sequenced and radio-labelled (Rediprime II, Amersham). After hybridisation membranes were washed with high stringency (0.1×SSC, 0.1%SDS, 65°C) and autoradiograms were obtained from a PhosphorImager (Fujifilm FLA3000).

### qPCR analysis

One microgram of total RNA was treated with DNase to remove genomic DNA and directly used to synthesize cDNA using the Quantitect kit (Qiagen). Out of the 20 μL of the reverse transcription final reaction volume, 1 μL was used as template for the qPCR reaction consisting of 0.4 μM of each primer and 1× SYBR green mastermix (Stratagene Brilliant kit or Eurogentec mesa fast qpcr mastermix plus for SYBR assays) in a final volume of 12.5 μL (using a Stratagene MX4000 Real Time thermocycler instrument and an Eppendorf ep realplex mastercycler). Serial dilutions of corresponding amplification product were used to monitor the amplification efficiency and to transform threshold cycles into concentrations. PCR conditions were 10 min at 95°C, then 40 cycles of 30 s at 95°C, 30 s at 58°C or 52°C, and 30 s at 72°C. Transcript levels were normalized to the expression level of *EF1a *(At5g60390) or *YLS8 *(At5g08290; [67]). Primer sequences are given in Additional file 5.

### Extraction and HPLC-MS^2 ^analysis of intact glucosinolates

Approximately 500 mg of fresh shoot and root material were frozen, ground in liquid nitrogen and freeze-dried. Samples (25-40 mg dry weight, DW) were extracted according to previously developed protocols [68]. GLS were identified and quantified by HPLC-PDA-MS^2 ^(LCQ Advantage, ThermoFinnigan). Separations were carried with a 250 × 4.6 mm 4 μm Synergi RP-Max column (Phenomenex, Maccesfield, UK) eluted at 1 ml/min with a 60 min gradient of 2-60% acetontrile in water containing 0.25% formic acid. After PDA detection, the column eluate was split and 0.2 ml/min directed to the electrospray interface of the mass spectrometer operating in negative ion mode and scanning from *m/z *250-750. Capillary temperature was 300°C, sheath and auxiliary gas were 60 arbitrary units, source voltage was 4.5 kV. The collision energy was optimized at 45 eV. The appearance of a MS^2 ^fragment ion at *m/z *97 was used as a characteristic fingerprint for GLS identification. Transitions used to identify 14 different GLS are presented in the Additional file 4. Individual GLS were quantified using single ion monitoring and the data obtained are presented as μmol/g dry weight of sinigrin equivalents.

## Authors' contributions

ST, WM and AC performed and analysed the glucosinolate measurements. TRL and IAG performed and analysed the oxylipin measurements. PA performed and analysed the transcript measurements. PA designed the experiments, integrated all data and wrote the first version of the manuscript. AA had overall responsibility for the project and wrote the final version of the manuscript. All authors contributed to the text, and read and approved the final version of the manuscript.

## Supplementary Material

Additional file 1 **Response of genes with a putative role in oxylipin biosynthesis to K-deficiency and re-supply**. Log2 ratios of transcript levels (treatemnt/control) in plants grown for 2 weeks on -K or control media (left), and after 6 hours of K re-supply to K-starved plants (control plant were re-supplied with Na instead of K, or with K-free medium). Increase and decrease in transcript level is marked in pink and green respectively. Three batches of plants were grown and treated independently, each box represents one replicate. For details see [6]. Click here for file

Additional file 2 **Composition of growth media**. Final concentrations (in mM) of macronutrients in growth media sufficient in all nutrients (control) or deficient in potassium (-K), nitrogen (-N), phosphorus (-P) or calcium (-Ca). For micronutrients see [65]. Click here for file

Additional file 3 **Phenotypes of nutrient deficient plants**. *A. thaliana *plants grown for 2 weeks on control medium or medium deficient in potassium (-K), nitrogen (-N), phosphorus (-P) or calcium (-Ca). For composition of growth media see Additional file 2. Click here for file

Additional file 4 **Names, abbreviations and transitions of glucosinolates determined in this study**. The appearance of a MS^2 ^fragment ion at *m/z *97 was used as a characteristic fingerprint for glucosinolate identification. Transitions used to identify 14 different glucosinolate are presented in the last column. Click here for file

Additional file 5 **Primer sequences for PCR**. Primer sequences used to amplify fragments of *A. thaliana *transcripts with putative roles in oxylipin or glucosinolate biosynthesis. Click here for file
