# Supplementary material for: Potassium deficiency induces the biosynthesis of oxylipins and glucosinolates in Arabidopsis thaliana
Source: BMC Plant Biol. 2010 Aug 11;10:172. doi: 10.1186/1471-2229-10-172 (PMC3017790; doi:10.1186/1471-2229-10-172)
Supplement: Additional file 1 — Response of genes with a putative role in oxylipin biosynthesis to K-deficiency and re-supply. Log2 ratios of transcript levels (treatemnt/control) in plants grown for 2 weeks on -K or control media (left), and after 6 hours of K re-supply to K-starved plants (control plant were re-supplied with Na instead of K, or with K-free medium). Increase and decrease in transcript level is marked in pink and green respectively. Three batches of plants were grown and treated independently, each box represents one replicate. For details see [6]. [file 1471-2229-10-172-S1.PDF]

JA biosynthesis genes expression profile in K starved and K resupplied A. thaliana plants

Wild-type Col0 shoots

AGI	Name	Description	K starvation			K(re-supply) +K for 6h					
			-K for 14Days			+ K (/Na)			+ K (/-K)		
AT1G55020	LOX1	lipoxygenase	-0.4	-0.3	-0.1	-0.1		0.2	0.2	-0.1	
AT3G45140	LOX2	lipoxygenase	2.9	1.6	1.7	-1.2	-1.1	-1.8	-1.2	-1.2	-1.1
AT1G17420	LOX3	lipoxygenase	0.1	0.3	0.4	-0.4	-0.3	-0.4	-1.0	-0.8	0.9
AT1G72520	LOX4	lipoxygenase	0.2	0.4	0.5		-0.4	-0.8	-0.8	-0.7	-0.7
AT3G22400	LOX5	lipoxygenase	0.4	0.2		0.1			-0.3	-0.6	-0.1
AT3G01420	AT3G01420	lipoxygenase	0.1	0.5	0.8	-0.4	-0.1	-0.7	-0.3	-0.5	
AT1G73680	AT1G73680	lipoxygenase	0.0	0.1	-0.1	-0.0	-0.2	-0.3	-0.2		
AT1G67560	AT1G67560	lipoxygenase		-0.2	-0.2	0.2	-0.1	0.2		0.0	-0.2
AT5G42650	AOS	allene oxide synthase	0.9	0.8	0.5	-1.2	-0.9	-1.5	-1.3	-1.3	-1.2
AT3G25760	AOC1	allene-oxide cyclase	1.1	1.5	1.1	-0.2	-0.5	-0.8	-1.1	-0.8	-1.0
AT3G25770	AOC2	allene oxide cyclase	-0.1	0.1			0.2	-0.1		0.2	
AT3G25780	AOC3	allene-oxide cyclase	0.1	0.7	0.4	-0.1	-0.5	-0.4	-0.9	-0.8	-0.5
AT1G13280	AOC4	allene-oxide cyclase	0.4	0.1	-0.2	-0.3	0.1	0.1	-0.2	-0.1	-0.4
AT2G06050	OPR3	12-oxophytodienoate reductase	0.6	0.1	0.2	-0.6	-0.6	-0.8	-0.8	-0.9	-0.3
AT1G76680	AT1G76680	12-oxophytodienoate reductase	-0.2	-0.3	0.1	0.1	-0.5	0.2	-0.2	0.1	-0.0
AT1G20510	OPCL1	3-oxo-2-(2'-[Z]-pentenyl)cyclopentane-1-octanoate CoA ligase	0.7	0.2	-0.1	-0.3		-0.2	-0.2	-0.3	-0.4
AT4G16760	AT4G16760	OPC6 acyl-CoA oxidase	0.4		0.3	-0.1	-0.2	0.3	-0.1		
AT4G29010	AT4G29010	OPC8-trans-2-enoyl-CoA hydratase	0.3	0.4	-0.1	0.1	-0.1	-0.3	-0.2	-0.3	
AT2G33150	KAT2	OPC8-3-ketoacyl-CoA thiolase	0.8	0.6	-0.1	-0.3	-0.2	-0.4	-0.4	-0.5	-0.4

Transcript expressed in log2 ratios vs control; see Armengaud et al. 2004
