# Supplementary material for: Potassium deficiency induces the biosynthesis of oxylipins and glucosinolates in Arabidopsis thaliana
Source: BMC Plant Biol. 2010 Aug 11;10:172. doi: 10.1186/1471-2229-10-172 (PMC3017790; doi:10.1186/1471-2229-10-172)
Supplement: Additional file 2 — Composition of growth media. Final concentrations (in mM) of macronutrients in growth media sufficient in all nutrients (control) or deficient in potassium (-K), nitrogen (-N), phosphorus (-P) or calcium (-Ca). For micronutrients see [65]. [file 1471-2229-10-172-S2.PDF]

**Composition of growth media**

| **Salt /mM** | **Control** | **-K** | **-N** | **-P** | **-Ca** |
|---|---|---|---|---|---|
| $KNO_3$ | 1.25 | - | - | 1.25 | 1.25 |
| $Ca(NO_3)_2$ | 0.5 | 1.0 | 0.2 | 0.5 | 0.1 |
| $MgSO_4$ | 0.5 | 0.5 | 0.5 | 0.5 | 0.5 |
| $KH_2PO_4$ | 0.625 | - | 0.625 | - | 0.625 |
| $NaH_2PO_4$ | - | 0.625 | - | - | - |
| NaCl | 2.0 | 1.375 | 1.0 | 1.375 | 1.2 |
| $CaCl_2$ | - | - | 0.3 | - | - |
| KCl | - | - | 1.25 | 0.625 | - |
| $MgCl_2$ | - | - | - | - | - |
| $NaNO_3$ | - | - | - | - | 0.8 |
| **Ion /mM** | | | | | |
| $K^+$ | 1.875 | **0.0** | 1.875 | 1.875 | 1.875 |
| $Ca^{2+}$ | 0.5 | 1.0 | 0.5 | 0.5 | **0.1** |
| $Mg^{2+}$ | 0.5 | 0.5 | 0.5 | 0.5 | 0.5 |
| $NO_3^-$ | 2.25 | 2.0 | **0.4** | 2.25 | 2.25 |
| $SO_4^{2-}$ | 0.5 | 0.5 | 0.5 | 0.5 | 0.5 |
| $PO_4^{3-}$ | 0.625 | 0.625 | 0.625 | **0.0**[2] | 0.625 |

[1] From micronutrients
