# Supplementary figures and images for: Potassium deficiency induces the biosynthesis of oxylipins and glucosinolates in Arabidopsis thaliana
Source: BMC Plant Biol. 2010 Aug 11;10:172. doi: 10.1186/1471-2229-10-172 (PMC3017790; doi:10.1186/1471-2229-10-172)

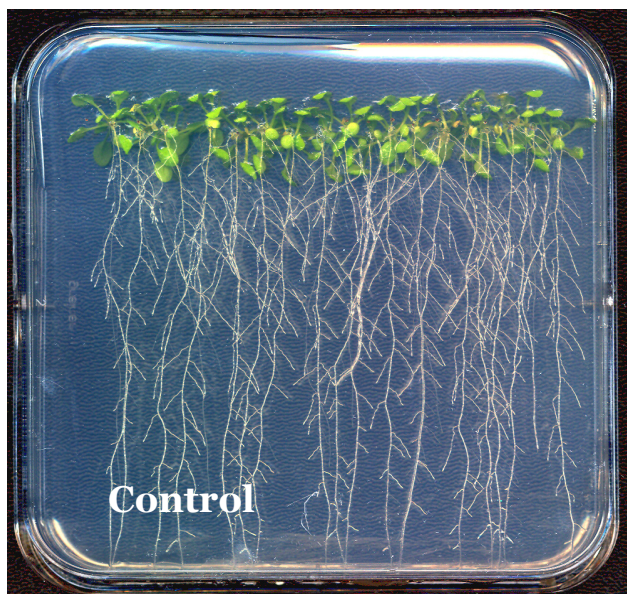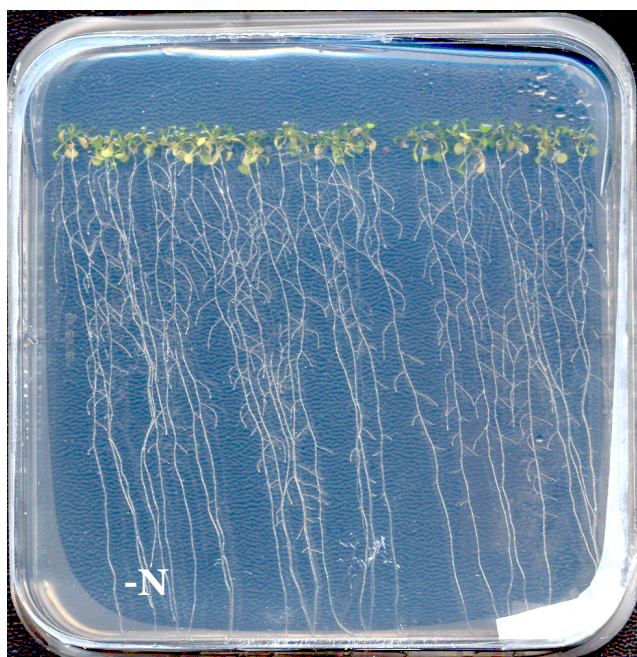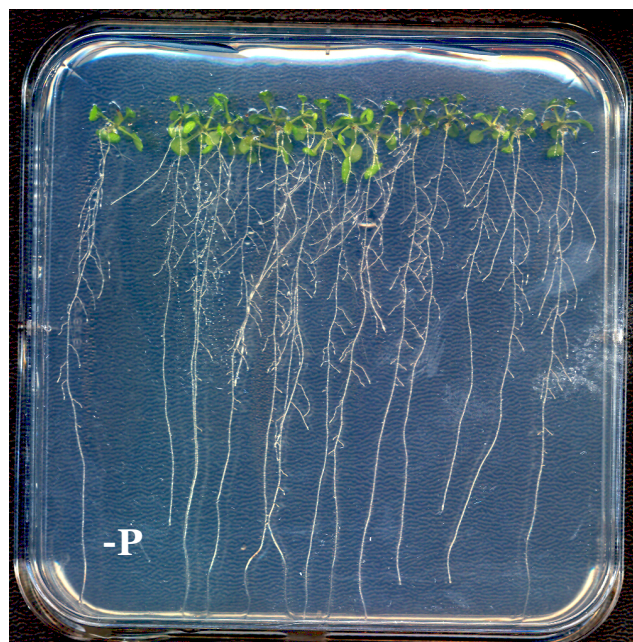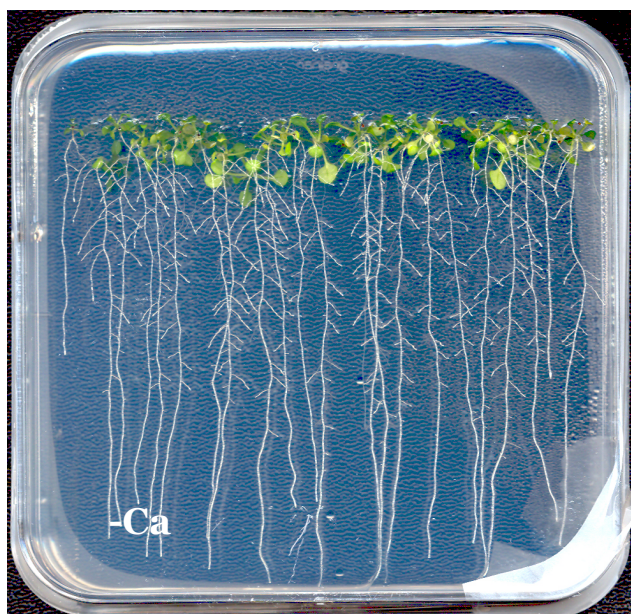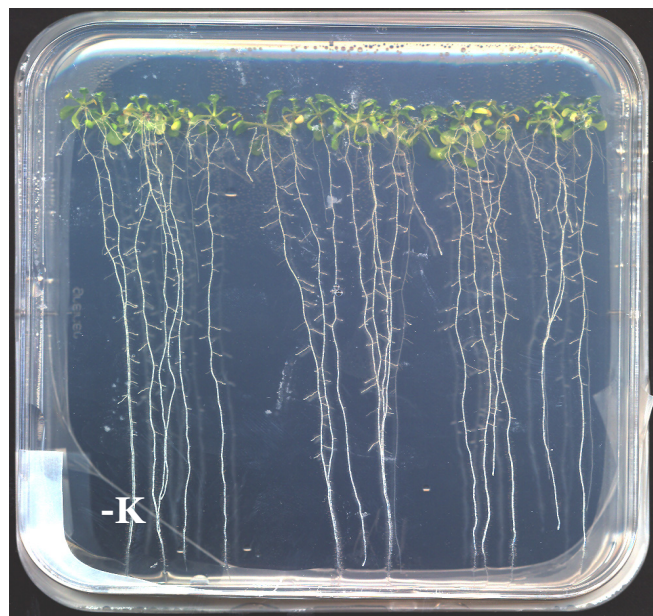

Supplement: Additional file 3 — Phenotypes of nutrient deficient plants. A. thaliana plants grown for 2 weeks on control medium or medium deficient in potassium (-K), nitrogen (-N), phosphorus (-P) or calcium (-Ca). For composition of growth media see Additional file 2. [file 1471-2229-10-172-S3.PDF]
