# Supplementary material for: Potassium deficiency induces the biosynthesis of oxylipins and glucosinolates in Arabidopsis thaliana
Source: BMC Plant Biol. 2010 Aug 11;10:172. doi: 10.1186/1471-2229-10-172 (PMC3017790; doi:10.1186/1471-2229-10-172)
Supplement: Additional file 4 — Names, abbreviations and transitions of glucosinolates determined in this study. The appearance of a MS2 fragment ion at m/z 97 was used as a characteristic fingerprint for glucosinolate identification. Transitions used to identify 14 different glucosinolate are presented in the last column. [file 1471-2229-10-172-S4.PDF]

Transitions used for determination of individual glucosinolates

Full name	Abbreviation	Transition
3-methylsulfinylpropyl	3MSOP	422>97
4-methylsulfinylbutyl	4MSOB	436>97
5-methylsulfinylpentyl	5MSOP	450>97
6-methylsulfinylhexyl	6MSOH	464>97
7-methylsulfinylheptyl	7MSOH	478>97
8-methylsulfinyloctyl	8MSOO	492>97
4-methylthiobutyl	4MTB	420>97
5-methylthiopentyl	5MTP	434>97
7-methythioheptyl	7MTH	462>97
8-methylthiooctyl	8MTO	476>97
indol-3-ylmethyl	I3M	447>97
4-hydroxy-indol-3-ylmethyl	4OHI3M	463>97
4-methoxy-indol-3-ylmethyl	4MOI3M	477>97
1-methoxy-indol-3-ylmethyl	1MOI3M	477>97
