# Supplementary material for: Potassium deficiency induces the biosynthesis of oxylipins and glucosinolates in Arabidopsis thaliana
Source: BMC Plant Biol. 2010 Aug 11;10:172. doi: 10.1186/1471-2229-10-172 (PMC3017790; doi:10.1186/1471-2229-10-172)
Supplement: Additional file 5 — Primer sequences for PCR. Primer sequences used to amplify fragments of A. thaliana transcripts with putative roles in oxylipin or glucosinolate biosynthesis. [file 1471-2229-10-172-S5.PDF]

Primer sequences for qPCR

Gene	Name	Forward primer 5'-	Reverse primer 5'-	Size (bp)
At3g45140	LOX2	TACGGATATGGAGGATACTTTC	CAGGTGAATGTGTTGATAAAAG	193
At5g24770	VSP2	ATATGGATACGGAACAGAGAAG	CCATTAGGCTTCAATATGAGAT	234
At4g39950	CYP79B2	AGGAAAGAGAACTCAAATCG	ATCTCTTCCATTGCTTTACG	210
At2g22330	CYP79B3	CTCTATCAAGGACGAAGCTG	TTGGTATATCGGATTCTTGG	213
At5g08290	YLS8	ACTGGGATGAGACCTGTATG	TGTTGTTGTTACCAGTTCCA	204
At3g45140	LOX2	TTAAAGGAAACATGACAGTTGA[1]	CAGGTGAATGTGTTGATAAAAG[1]	1115
At5g24770	VSP2	TCCTCTCACTTTCACTTCTCTT[1]	CCATTAGGCTTCAATATGAGAT[1]	625
At1g55020	LOX1	TAAACACTACACTACAGAC	CATAGTCATATACTCTGTC	158
At1g17420	LOX3	AGTATATTGGAGAGAGAC	TAGATACACTATTAGGTACA	231
At5g42650	AOS	GTGAAATGCTTTACGGTTATC	ACCAAAACAACAAAATCCTTAC	200
At3g25760	AOC1	TTCTACACTTTTTATCTTA	TTATTATATATACAGGACAC	193
At2g06050	OPR3	CCATAGGAGCTAGTAAAGTTGG	CCACTAGACATAAAGGTTCCAT	277
At1g16410	CYP79F1	TTTCTATGTTATGTTACTAC	TACTCTAGTCCTAAACAA	203
At1g16400	CYP79F2	AATATCGTAGTGTTCATA	CTAGTAAAGTGATTGATG	153
At2g20610	SUR1	TATAGAAAGAAGAACTCA	GTAGATGACACCTCTAAG	195
At4g31500	SUR2	GCTATATATATCATTAGGA	ACTACTTTCTTTTCTAGTC	160
At1g74100	ST5a	GAGAAATTCAAAGATACT	TAAATAAGATGATACAGAC	150
At5g60390	EF1a	CTGGAGGTTTTGAGGCTGGTAT	CCAAGGGTGAAAGCAAGAAGA	72

[1]RT-PCR for Northern probes
